# A Single Pelvic Fibrous Tumor Associated With Doege–Potter Syndrome: A Case Study

**DOI:** 10.1155/2024/4584292

**Published:** 2024-10-14

**Authors:** Wangjia Mao, Yuefang Sun, Shan Yan, Yuan Gao, Le Bu, Xiaoyun Cheng

**Affiliations:** ^1^Department of Endocrinology and Metabolism, Shanghai Tenth People's Hospital, School of Medicine, Tongji University, Shanghai, China; ^2^Department of Pathology, Shanghai Tenth People's Hospital, Tenth People's Hospital of Tongji University, 301 Yanchang Middle Road, Shanghai 200072, China; ^3^School of Medicine, Tongji University, Shanghai 200092, China; ^4^Department of Endocrinology, Guangrao County People's Hospital, Dongying 257300, China; ^5^Department of Endocrinology, Chongming Branch of Shanghai Tenth People's Hospital, Shanghai, China

**Keywords:** Doege–Potter syndrome (DPS), hypoglycemia, IGF-Ⅱ, laparoscopic retroperitoneal, pelvic, solitary fibrous tumors (SFTs)

## Abstract

Doege–Potter syndrome (DPS) is a very rare paraneoplastic condition that is marked by hypoglycemia brought on by a solitary fibrous tumor rather than an islet cell tumor. Soft tissue neoplasms termed as solitary fibrous tumors (SFTs) are rare and these tumors vary in the site of origin, from the pleural cavity, mediastinum, pericardium, retroperitoneal spaces, liver, thyroid, orbit, bladder, intestines, and soft tissues, while pelvic-derived fibrous tumors are incredibly unusual. There are currently extremely few documented cases and literature reviews both domestically and internationally. In this case study, we present an 82-year-old woman who developed DPS as a result of malignant pelvic SFTs. Her hypoglycemia was clinically healed after she underwent laparoscopic retroperitoneal tumor resection in our institution, and thereafter, her quality of life improved.

## 1. Introduction

Solitary fibrous tumors (SFTs) were reported for the first time by Wagner et al. in 1870, and Klemperer and Rabin later detailed its histopathology findings in 1931 [1]. The most common location for SFTs is the thoracic cavity, although these SFTs are typically asymptomatic and only inadvertently discovered. The second most typical location for SFT is the abdominal cavity, which is followed by less typical sites such the trunk, head and neck, and extremities [2]. However, some SFTs have the potential to become malignant; the majority of SFTs are benign lesions that progress slowly [3]. In 1930, Doege and Potter first described non-islet cell tumor hypoglycemia (NICTH) [4]. Doege–Potter syndrome (DPS), known to be a uncommon and challenging paraneoplastic illness, is caused by the NICTH that occurs in almost 4%–11.5% of SFTs patients [4, 5]. The predominant symptom of DPS is severe and refractory hypoglycemia, which can occasionally be life-threatening. Consistent hypoglycemia, suppressed blood insulin level, C-peptide, and low serum insulin-like growth factor-II (IGF-II) levels, in contrary to normal or high levels of IGF-II, are the hallmarks of the DPS [6]. Complete tumor resection has usually been the mainstay of SFT treatment since SFTs are often well-circumscribed masses that grow expansively rather than invasively.

Here, we present a case of pelvic SFT with DPS that was successfully managed with surgical resection.

## 2. Case Presentation

An 82-year-old woman was referred to our hospital for further evaluation and treatment after she experienced episodic loss of consciousness for 11 months and remained repeatedly unconscious with hypoglycemia in the morning. She had no history of diabetes or other-related conditions. Upon endocrinological assessment at admission, a lab assessment including tumor markers, revealed no abnormalities other than hypoglycemia (2.04 mmol/L) and suppressed serum insulin (<0.2 mU/L) and C-peptide (0.08 ng/ml) but no anti-insulin antibodies. The results of oral glucose tolerance showed severe hypoglycemia, inhibition of insulin secretion, and decrease of C-peptide. Although it shows hypoglycemia in 10% of the normal individuals.

While performing a computed tomography (CT) scan, it was discovered that the bladder, uterus, rectum, and other adnexal structures were being compressed by a large solid mass in the pelvic area that was almost 10 cm in diameter and had heterogenous enhancement.

Contrast-enhanced CT demonstrated that the lesion's enhancement amplitude extended with time and the density eventually became uniform, exhibiting a “fast in slow out” kind of enhancement. Although several of the nearby organs were compressed, no obvious invasion of the bladder, uterus, pelvic sidewall, or rectum was found. There were no visible lymphadenopathy or other metastatic disease symptoms (data not shown).

A well-defined big cystic-solid mass measuring 10.6 cm × 9.9 cm × 10.8 cm was visible on magnetic resonance imaging (MRI), with iso-signal on T1 weighted image (T1WI), slightly low signal on T2 weighted image (T2WI), and a definite high signal on diffusion-weighted imaging (DWI). After enhancement, there was a significant decrease in apparent diffusion coefficient (ADC) value and a moderate enhance in solid components.

**Figure 1:**
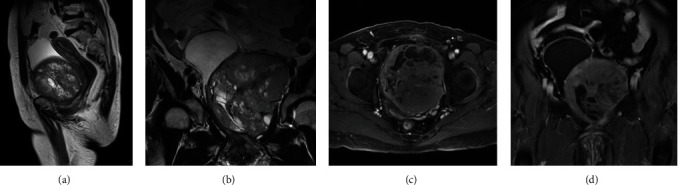
MRI at presentation. (A) T1WI MRI showed a low-intensity mass; (B) MRI showed the mass oppresses the bladder; (C and D) cystic signals of varying sizes on the mass. MRI, magnetic resonance imaging; T1WI, T1 weighted image.

The cystic structure was dispersed in the form of cystic foci of varying sizes, predominantly in the lesion's periphery, with low signal intensity on T1WI and high signal intensity on T2WI, and no unusual augmentation after enhancement was observed. The muscles in front of the lesion and the abdominal wall were not completely delineated. Prolific compression and displacement of the urinary bladder and uterus were observed, resulting in poor urinary bladder filling as well as compression of the lower segment of the left ureter, aiding in dilatation and effusion of the proximal ureter (Figure 1).

**Figure 2:**
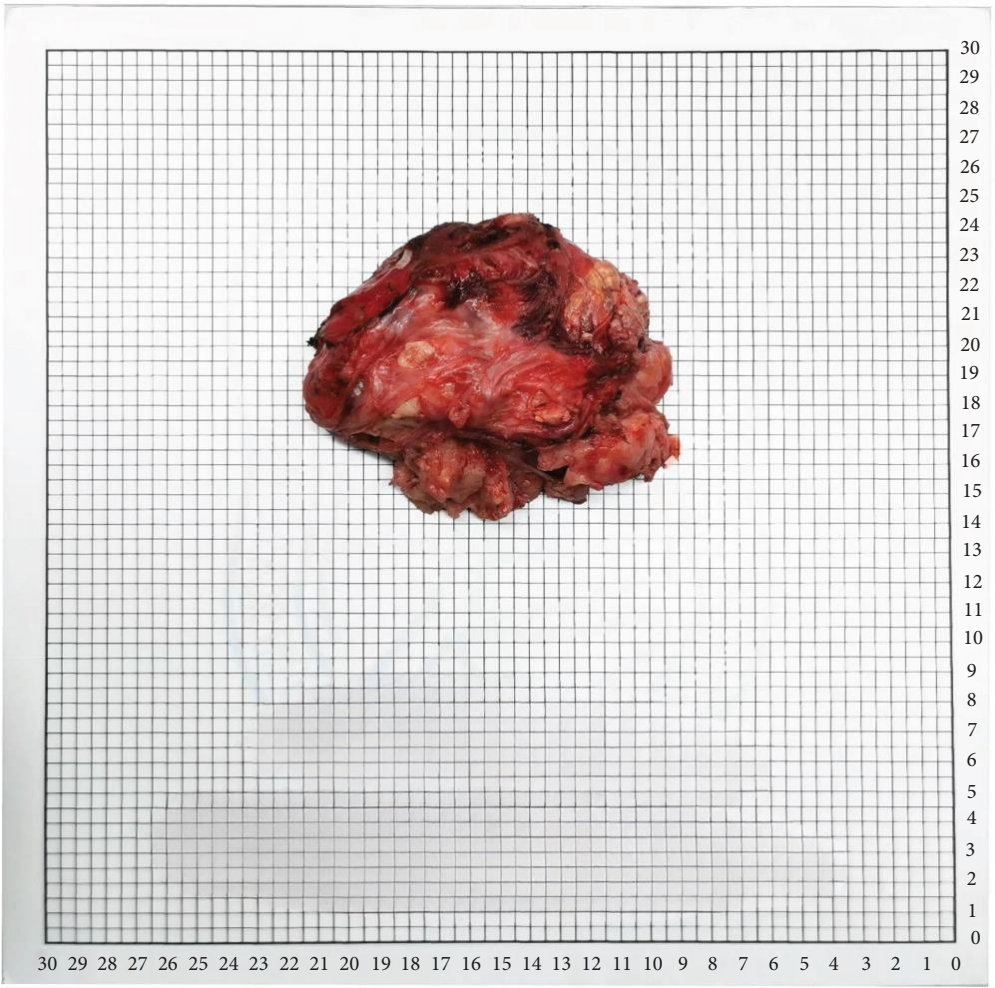
Macroscopic appearance of the tumor.

As a favored method of treatment, reasonable surgical treatment should always be kept in mind in tumors of insidious source to obtain optimum regional and systemic significant improvement whilst still maintaining patient's overall functions. Therefore, laparoscopic retroperitoneal lesion resection was carried out, and the 12 cm × 10 cm × 8 cm tumor was excised during the procedure (Figure 2).

**Figure 3:**
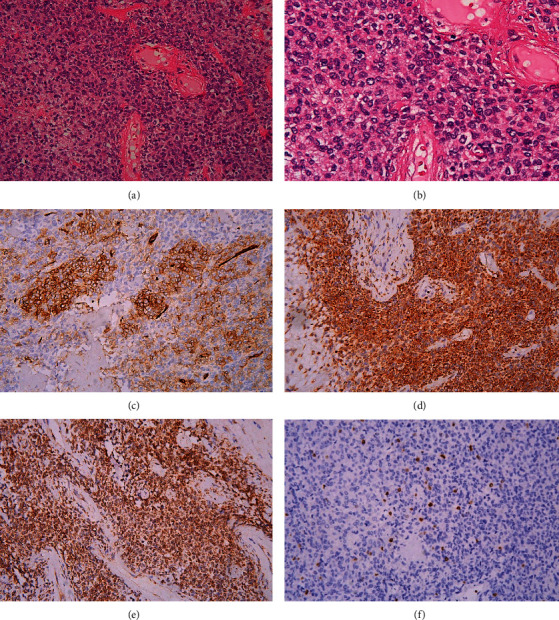
Pathological findings. H&E staining of the tumor showed monotonous spindle cell proliferation without atypia (A) 2/10 HPFs; (B) 4/10 HPFs). The mitosis rate was low (2/10 HPFs). Immunohistochemical staining demonstrated that the tumor cells were positive for (C) CD34, (D) Bcl-2, (E) STAT6, and (F) Ki67, consistent with a diagnosis of SFT. Bcl-2, B-cell lymphoma-2; CD34, cluster of differentiation 34; H&E, hematoxylin and eosin; HPFs, high-power fields, Ki67, Kiel 67; STAT6, signal transducer and activator of transcription 6.

Later, the excised mass was sent for pathological examination, and the results showed that the tumor cells were spindle shaped under a light microscope and had a patternless arrangement. The tumor cells showed severe atypia with focal necrosis, and the mitosis index was more than four mitosis per 10 high-power fields (HPFs). Immunohistochemistry (IHC) revealed that the specimen was cluster of differentiation 34-positive (CD34^+^), Bcl-2 positive, and nuclear positivity for signal transducer and activator of transcription 6 (STAT6) and the Kiel 67 (Ki67) index was 15% in the main tumor but negative for alpha-smooth muscle actin (SMA), AE1/AE3, desmin, sex determining region Y-box 2 (SOX2), transducin-like enhancer of split 1 (TLE-1), sal-like protein 4 (SALL4), S100, and claudin 4. These results unmistakably support the accurate interpretation of this pelvic tumor as SFTs as opposed to leiomyoma (Figure 3).

**Figure 4:**
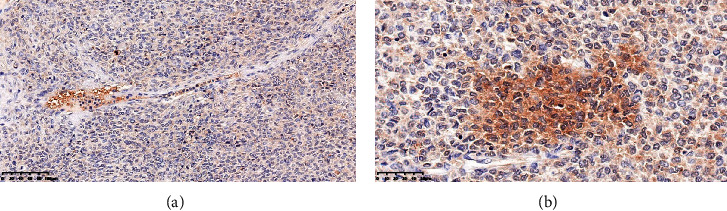
Immunohistochemical staining of IGF-II. (A) The bar represents 100 μm. (B) The bar represents 50 μm. IGF-II, insulin-like growth factor-II.

As was previously mentioned, the patient had nocturnal hypoglycemic symptoms while having low blood insulin levels; the current pelvic tumor was unquestionably the SFT because it produced IGF-II, which led to hypoglycemia. We carried out high-throughput IGF-II staining to confirm the current conclusion, and the results showed that it was in fact SFT rather than any other tumors (Figure 4).

## 3. Discussion

SFTs can generally happen anywhere on the body. As per the data analysis of National Cancer Institute of the United States of more than 40 years, the patients with involvement of the chest, abdomen, or pelvic cavity were 35.3%, central nervous system involvement accounted for 24.5%, and head, neck, or limb accounted for 19.9% and 16.2% of patients, respectively. However, only a small number of studies have documented occurrences of primary SFTs affecting the pelvic region [7].

We hereby reported a very rare case of SFT of pelvic origin with DPS. It confirmed that the tumor cells had produced and secreted IGF-II and that the serum IGF-II had dramatically decreased after tumor resection. The metabolic pathways activated by IGF-II involve insulin receptor (IR) and IGF receptor-I. The activation of IRs inhibits both hepatic gluconeogenesis and glycogenolysis and increases glucose uptake in muscle cells. In NICTH, hypoglycemic effects are predominantly mediated through unprocessed IGF-II, also known as big IGF-II. Since it has been found that the secretion of mature IGF-II from the tumor is relatively low compared to big IGF-II, possibly due to the generally high upregulation of IGF-II expression exceeding the cell's capacity for posttranslational processing. 80%–90% of processed IGFs are found in conjunction with IGF binding proteins (IGFBPs), forming ternary complexes that cannot cross capillary membranes, limiting IGFs' interactions with IRs [8].

Rarely occurs in the retroperitoneal location. In the literature, fewer than 30 cases have been reported [9]. SFTs usually affect midadults in both genders. In this case, she is an 82-year-old woman, so it is extremely rare. There was no symptom in thoracic SFTs but in extrathoracic SFTs. Clinical manifestation depends on the location of tumor. It has been reported that around 5% of SFTs are associated with hypoglycemia due to the secretion of IGFs [10]. Interestingly, the case we reported happened to be DPS secondary to SFT, secreting IGF-II led to intractable hypoglycemia.

SFTs are histologically comprised of spindled to ovoid cells with unclear, pale eosinophilic cytoplasm within of a variably collagenous stroma, and they can mimic numerous unrelated mesenchymal and nonmesenchymal tumor types. In SFTs, CD34 is one of the most distinctive classical IHC markers. Tumor induced hypoglycemia (TIH) was mainly thought due to humoral consumption of glucose, until the elucidation of “big-IGF-II” as etiology of most NICTH cases, as elaborated by Daughaday et al. The underlying mechanism of hypoglycemia in SFT is fusion of NGFI-A–binding protein 2 (NAB2)–STAT6 resulting in over expression of IGF-II, unprocessed resulting in larger molecular weight, unbound by IGFBP, acting on IGF-I and IGF-II receptors, but also IR resulting in hypoglycemia. NAB2–STAT6 induced expression of IGF-II can be inhibited by steroids, thus glucocorticoids is another mainstay of inoperable cases, with anecdotal cases of sue of phosphoinositide 3-kinases (PI3K) inhibitors that act downstream of IR signaling [11–13]. The most reliable and precise IHC marker of SFTs was recently shown to be the robust nuclear STAT6 expression driven on by a gene fusion called NAB2–STAT6 [14]. Bcl-2 and vimentin are typically immunoreactive in SFTs in addition to CD34 and STAT6, but SMA and desmin (a marker for myocytes), S-100 and synaptophysin (a marker for perineural tissue), or c-kit (a marker for gastrointestinal stromal tumors) are generally negative [4, 15, 16]. The majority of SFTs are benign, but some may invade local structures or metastasize.

DPS is a paraneoplastic syndrome associated with SFTs, and its predominant symptom is severe and refractory hypoglycemia. In patients with DPS, neoplastic cells of SFTs overexpress and secret high molecular weight (HMW)-IGF-II, which is insufficiently processed into IGF-II protein, resulting in NICTH [16, 17].

Through a retrospective examination of reported instances of cancer-related hypoglycemia, Scalia et al. [18] and his team discovered that the vast majority (95%, or 171 cases out of 180) of the associated IGF was attributed to secreted IGF-II. In summary, the prevalence of cancers exhibiting hypoglycemic symptoms that are also associated with the secretion of IGF-II far surpasses the quantity and diversity of tumors linked to IGF-I expression or secretion, which aligns with the observations made in this particular case study. IGF-II production is apparently correlated with tumor growth, and IGF-II overexpression is seen in more than 90% of tumors larger than 9 cm. The mainstay of SFT treatment has been surgical resection. For DPS, there are many treatments available. Amongst all, continuous intravenous glucose infusion and medical therapy are beneficial, which belong to short-term therapy [19]. Other treatments include adjuvant chemotherapy and radiotherapy [20, 21]. But single chemotherapy or radiotherapy is ineffective in treating tumors and severe hypoglycemia caused by tumors. SFT has traditionally been treated with surgical resection. Complete tumor resection is typically possible because SFTs grow expansively rather than invasively and are well-circumscribed masses [22]. In our case, the pelvic tumor (10.6 × 9.9 × 10.8) was so large that it had strong adhesions to the rectum and the left ureter. So laparoscopic intestinal adhesion loosening and laparoscopic retroperitoneal lesion resection were chosen. So far, it turns out that surgical resection is the most effective treatment with low recurrence rate, cure, or significant relief of hypoglycemia.

In 2013, based on the World Health Organization (WHO) classification of soft tissue and bone tumors, SFTs were considered benign tumors with the potential for malignant transformation [23]. Most SFTs are inert without local or distant recurrence. Still, some SFTs can be considered malignant with the following histopathologic characteristics: cytological atypia, hypercellularity, tumor necrosis, high mitotic rate (>4 per HPF), and/or infiltrative margins [24].

Furthermore, it is well documented that SFTs with DPS are substantially more malignant than SFTs without the syndrome. Han et al. [4] reported that up to 60% of SFTs with the DPS were malignant, as comparison to just 5%–10.4% of those without the condition.

In our case scenario, surgical excision of the mass was crucial because the patient had a massive SFT tumor exceeding 10 cm in size with DPS. Statistics show that the patient's quality of life and hypoglycaemic coma symptoms greatly improved after the pelvic mass was surgically removed.

## 4. Conclusion

We presented a very uncommon case of pelvic-originated SFT with DPS. We have established that the IGF-II that the tumor was producing was what triggered the distinct hypoglycemic bouts to occur. Following the surgical removal of the tumor, those symptoms reduced dramatically. Short-term and long-term follow-ups are crucial for monitoring the recurrence and metastasis of these SFT since they are very prone to recurrence.

## Data Availability

Research data are not shared.
